# Heregulin (HRG) assessment for clinical trial eligibility testing in a molecular registry (PRAEGNANT) in Germany

**DOI:** 10.1186/s12885-020-07546-1

**Published:** 2020-11-11

**Authors:** Hanna Huebner, Christian M. Kurbacher, Geoffrey Kuesters, Andreas D. Hartkopf, Michael P. Lux, Jens Huober, Bernhard Volz, Florin-Andrei Taran, Friedrich Overkamp, Hans Tesch, Lothar Häberle, Diana Lüftner, Markus Wallwiener, Volkmar Müller, Matthias W. Beckmann, Erik Belleville, Matthias Ruebner, Michael Untch, Peter A. Fasching, Wolfgang Janni, Tanja N. Fehm, Hans-Christian Kolberg, Diethelm Wallwiener, Sara Y. Brucker, Andreas Schneeweiss, Johannes Ettl

**Affiliations:** 1 Department of Gynecology and Obstetrics, Comprehensive Cancer Center Erlangen-EMN, Erlangen University Hospital, Friedrich-Alexander University Erlangen-Nuremberg, Universitaetsstrasse 21-23, Erlangen, 91054 Germany; 2 Gynecology I (Gynecologic Oncology), Gynecologic Center Bonn-Friedensplatz, Bonn, Germany; 3 Merrimack Pharmaceuticals, Cambridge, MA USA; 4 Department of Obstetrics and Gynecology, University of Tübingen, Tübingen, Germany; 5 Klinik für Gynäkologie und Geburtshilfe Frauenklinik St. Louise, Paderborn, St. Josefs-Krankenhaus, Salzkotten, Kooperatives Brustzentrum, Paderborn, Germany; 6 Department of Gynecology and Obstetrics, Ulm University Hospital, Ulm, Germany; 7 Ansbach University of Applied Sciences, Ansbach, Germany; 8 Department of Gynecology, Zurich University Hospital, Zurich, Switzerland; 9 Oncologianova GmbH, Recklinghausen, Germany; 10 Oncology Practice at Bethanien Hospital Frankfurt, Frankfurt, Germany; 11 Biostatistics Unit, Department of Gynecology and Obstetrics, University Hospital Erlangen, Erlangen, Germany; 12 Berlin, Campus Benjamin Franklin, Department of Hematology, Oncology and Tumor Immunology, Charité University Hospital, Berlin, Germany; 13 Department of Obstetrics and Gynecology, University of Heidelberg, Heidelberg, Germany; 14 Department of Gynecology, Hamburg-Eppendorf University Medical Center, Hamburg, Germany; 15 ClinSol GmbH & Co KG, Würzburg, Germany; 16 Department of Gynecology and Obstetrics, Helios Clinics Berlin Buch, Berlin, Germany; 17 Department of Gynecology and Obstetrics, University Hospital Düsseldorf, Düsseldorf, Germany; 18 Department of Gynecology and Obstetrics, Marienhospital Bottrop, Bottrop, Germany; 19 National Center for Tumor Diseases and Department of Gynecology and Obstetrics, Heidelberg University Hospital, Heidelberg, Germany; 20 Department of Obstetrics and Gynecology, Klinikum rechts der Isar, Technical University of Munich, Munich, Germany

**Keywords:** Advanced breast cancer, Metastatic, Antihormone therapy, Heregulin, Seribantumab, MM-121

## Abstract

**Background:**

Eligibility criteria are a critical part of clinical trials, as they define the patient population under investigation. Besides certain patient characteristics, clinical trials often include biomarker testing for eligibility. However, patient-identification mostly relies on the trial site itself and is often a time-consuming procedure, which could result in missing out on potentially eligible patients. Pre-selection of those patients using a registry could facilitate the process of eligibility testing and increase the number of identified patients. One aim with the PRAEGNANT registry (NCT02338167) is to identify patients for therapies based on clinical and molecular data. Here, we report eligibility testing for the SHERBOC trial using the German PRAEGNANT registry.

**Methods:**

Heregulin (HRG) has been reported to identify patients with better responses to therapy with the anti-HER3 monoclonal antibody seribantumab (MM-121). The SHERBOC trial investigated adding seribantumab (MM-121) to standard therapy in patients with advanced HER2-negative, hormone receptor–positive (HR-positive) breast cancer and HRG overexpression. The PRAEGNANT registry was used for identification and tumor testing, helping to link potential HRG positive patients to the trial. Patients enrolled in PRAEGNANT have invasive and metastatic or locally advanced, inoperable breast cancer. Patients eligible for SHERBOC were identified by using the registry. Study aims were to describe the HRG positivity rate, screening procedures, and patient characteristics associated with inclusion and exclusion criteria.

**Results:**

Among 2769 unselected advanced breast cancer patients, 650 were HER2-negative, HR-positive and currently receiving first- or second-line treatment, thus potentially eligible for SHERBOC at the end of current treatment; 125 patients also met further clinical eligibility criteria (e.g. menopausal status, ECOG). In the first/second treatment lines, patients selected for SHERBOC based on further eligibility criteria had a more favorable prognosis than those not selected. HRG status was tested in 38 patients, 14 of whom (36.8%) proved to be HRG-positive.

**Conclusion:**

Using a real-world breast cancer registry allowed identification of potentially eligible patients for SHERBOC focusing on patients with HER3 overexpressing, HR-positive, HER2-negative metastatic breast cancer. This approach may provide insights into differences between patients eligible or non-eligible for clinical trials.

**Trial registration:**

Clinicaltrials, NCT02338167, Registered 14 January 2015 - retrospectively registered.

**Supplementary Information:**

**Supplementary information** accompanies this paper at 10.1186/s12885-020-07546-1.

## Background

The recruitment of patients based on inclusion and exclusion criteria is an important determinant of efficient and successful clinical trials. Strict eligibility criteria are often necessary to reach the primary endpoint but simultaneously decrease the number of eligible patients and could result in poor recruitment rates [1]. Recent data report that 40% of all trials fail to reach their intended enrolment numbers despite general approaches to improve recruitment of eligible patients [2]. This could result in reduced statistical power due to low sample size and a delay and financial burden for the investigators and sponsors due to extended study durations [1]. To facilitate the recruitment process of clinical trials, registries can be a suitable tool in order to retrospectively identify potentially eligible patients [3]. Central matching of patients enrolled in the registry with the inclusion and exclusion criteria of a clinical trial decreases the amount of time for patient selection and could increase the number of patients identified. Besides selection based on known clinical patient and tumor characteristics, biomarker testing for eligibility is often an essential part of the selection process. In most of the cases, tissue for biomarker testing is collected locally and tested centrally. However, necessary sample collection and processing procedures have to be established at each study side. Central collection, processing and testing of biomaterial using data and biomaterial collected by a registry study could improve the time and process management and allow batched sample testing in a timely fashion [4].

In breast cancer (BC) patients, human epidermal growth factor receptors (HERs) have been identified as important drivers of pathogenesis and progression and can function as biomarkers for HER-directed therapies [5–8]. HER-directed therapies such as trastuzumab, lapatinib, neratinib, pertuzumab, ado-trastuzumab emtansine, and recently trastuzumab deruxtecan and tucatinib have shown efficacy in both metastatic and early BC [9–22]. Although the relevance of HER3 in patients with HER2-positive BC has been established through the introduction of effective therapies into the clinical routine, its role in HER2-negative patients is not yet fully understood. HER3 has been implicated in resistance to hormonal therapies and could be important in overcoming resistance to endocrine treatment. In preclinical studies, it has been shown that HER3 signaling may be involved in the resistance mechanisms of BC cell lines that are being treated with fulvestrant [23, 24]. In view of these facts, HER3 is currently the focus for several therapeutic agents that target it [25].

Overexpression of the main soluble ligands of HER3, neuregulins or heregulins (NRG/HRG), appears to identify a population who could benefit specifically from inhibition with an anti-HER3 antibody [26, 27]. Retrospective analyses of clinical studies in different cancer entities has shown that subpopulations with HRG-positive, HER2-negative cancers (ovarian, breast, and lung cancer) have a significantly longer progression-free survival (PFS) when treated with an anti-HER3 antibody (seribantumab, MM-121) in comparison with biomarker-negative patients [27, 28]. However, although HRG positivity was found to be predictive for benefiting from seribantumab therapy, HRG-positive patients in the control arm had more rapid progression in comparison with HRG-negative patients [27–29].

In the present study, a patient selection algorithm was applied in an established BC registry for patients with advanced BC, using the inclusion and exclusion criteria of a phase 2 study (SHERBOC) with the anti-HER3 antibody seribantumab (NCT03241810 and 2017–000565-76). Patients identified as eligible based on HRG expression and SHERBOC inclusion and exclusion criteria, were intended for enrollment into the trial. The primary study aim was to identify the rate of HRG-positive tumors in this patient population. In addition, the effect of patient selection on patient and tumor characteristics was analyzed.

## Methods

### The PRAEGNANT research network

The PRAEGNANT study (Prospective Academic Translational Research Network for the Optimization of the Oncological Health Care Quality in the Adjuvant and Advanced/Metastatic Setting; NCT02338167 [30]) is an ongoing, prospective BC registry with documentation methods similar to those of a clinical trial. Inclusion criteria for the advanced/metastatic setting are as follows: Adult women aged ≥18 years, patients with the diagnosis of invasive BC, patients, who are willing and able to sign the informed consent form and patients with metastatic or locally advanced, inoperable disease proven by clinical measures (i.e. standard imaging). The exclusion criteria are: Patients who did not sign the informed consent form and patients who are not eligible for observation due to non-availability and/or severe comorbidities as evaluated by the treating physician. Due to the limited inclusion/exclusion criteria, the population of the PRAEGNANT study is quite representative to the general population in regard to advanced or metastatic BC cases. Fifty-three study sites are part of the PRAEGNANT study. The aims of PRAEGNANT are to assess both treatment patterns and quality of life and to identify patients who may be eligible for clinical trials or specific targeted treatments, as well [30–33]. Patients can be included at any time point during the course of their disease. All patients provided informed consent. The consent procedure included the information that the disease, tumor characteristics and biomaterial are used for both scientific projects and eligibility assessments for clinical trials and other treatment opportunities. The study was approved by the relevant ethics committees.

### The SHERBOC trial

The SHERBOC Trial (MM-121-02-02-10; EudraCT number: 2017–000565-76; US: NCT03241810) was a randomized phase 2 trial comparing fulvestrant with fulvestrant + seribantumab (MM-121) in BC patients with advanced HER2-negative, hormone receptor–positive disease [34, 35]. Seribantumab (MM-121) is a fully human, monoclonal IgG2 antibody that binds to the ligand-binding domain of HER3 and inhibits HRG-mediated signaling [34]. Subjects were prospectively selected using an HRG RNA in situ hybridization assay. The primary end point of the SHERBOC trial wasPFS [34]. Detailed inclusion criteria are shown in Additional Table 1 [35]. The international trial was intended for screening of approximately 200 women globally to enroll 80 HRG positive subjects at roughly 80 sites. The actual global enrollment was 22 participants. In Germany, screening of patients was supported by the PRAEGNANT registry. In this report we focus on the German PRAEGNANT cohort. Ten of the 53 PRAEGNANT study sites were approved by the sponsor for SHERBOC and two were under review by the sponsor before termination of the trial. One of the approved sites was activated. The study was terminated by the sponsor following failure of seribantumab (MM-121) in a companion Phase 2 randomized study (SHERLOC) in HRG-positive lung cancer patients [36]. Thus, no patients from PRAEGNAT have been actually enrolled in SHERBOC.

### Computerized phenotyping of patients from the PRAEGNANT database

A script was created that extracted all the necessary variables using structured query language (SQL) statements from the original patient data in the PRAEGNANT database and determined eligibility status when executing the script.

### Patients

All patients were enrolled in the PRAEGNANT registry study and have signed the informed consent form. A survival analysis in this population was conducted for the therapy line before a possible study inclusion (PFS_-1TL_; see below).

### Data collection

Data were collected by trained staff and documented in an electronic case report form [30]. The data were monitored using automated plausibility checks and on-site monitoring. Data not usually documented as part of routine clinical work were collected prospectively using structured questionnaires on paper. These data comprised epidemiological data, including family history, cancer risk factors, quality of life, nutrition and lifestyle items, and psychological health. Additional Table 2 provides an overview of the collected data.

### Definition of hormone receptor, HER2 status, and grading

The definitions of hormone receptors, HER2 status, and grading have been described previously [31]. Data on estrogen receptor (ER) status, progesterone receptor (PR) status, HER2 status, and grading were required for documentation from each tumor biopsy. There could therefore be several sources for a single patient (right breast, left breast, local recurrence, metastatic site). Biomarker status for ER, PR, and HER2 were determined as follows: If a biomarker assessment of the metastatic site was available, this receptor status was taken for this analysis. If there was no information from metastases, the latest biomarker results from the primary tumor were taken. All patients treated with endocrine therapy (ET) were assumed to be hormone receptor–positive, and all patients who had ever been treated with anti-HER2 therapy were assumed to be HER2-positive. There was no central review of these biomarkers. The study protocol recommended assessing ER and PR status as positive if ≥1% was stained. A positive HER2 status required an immunohistochemistry (IHC) score of 3+ or positive fluorescent in situ hybridization/ competitive in situ hybridization (FISH/CISH).

### HRG testing

HRG analysis was performed on formalin-fixed, paraffin-embedded (FFPE) sections from the latest available biopsy. PRAEGNANT samples from patients who were potentially eligible for the SHERBOC trial were either consolidated and sectioned in a central screening facility with the PRAEGNANT network or at the study sites themselves. Both screening mechanisms were allowed. HRG mRNA expression levels were analyzed using a validated chromogenic-based RNA in situ hybridization (RNA-ISH) assay performed by a central laboratory (Covance Laboratories, Burlington, North Carolina, USA) with College of American Pathologists (CAP) and Clinical Laboratory Improvement Amendments (CLIA) certification, using reagents and technology developed by Advanced Cell Diagnostics (Newark, California, USA) on a Leica (Buffalo Grove, Illinois, USA) Bond Rx autostainer. Slides were evaluated by a board-certified pathologist as follows:

- Cancer cell content assessment, excluding any slides with < 50 cancer cells in total
- Scoring of cancer cells: 0, 0 RNA-ISH dots; 1, 1–3 dots per tumor cell; 2, > 4 dots per tumor cell
- Overall HRG expression was computed as the highest score in at least 10% of tumor cells

Run and tissue controls were included to ensure the integrity of the process, RNA quality and limited nonspecific binding of probes/ reagents. Samples that met the control criteria and scored ≥1 were considered HRG-positive.

### Statistical analysis

Patients and tumor characteristics grouped by SHERBOC status were described using summary statistics. SHERBOC status (yes) was defined as patients meeting all SHERBOC criteria – without HRG status – as documented in the PRAEGNANT registry and who would be potentially eligible for inclusion if tested HRG positive. Mean and standard deviation (SD) are shown for continuous characteristics, and frequency and percentage for categorical characteristics.

Patients selected for SHERBOC at the next progression were compared with patients who were not selected for SHERBOC in relation to the prognosis. For this purpose, PFS before inclusion (− 1 therapy line, −1TL; PFS_–1TL_) was defined from the date of previous therapy initiation to the earliest date of disease progression (distant metastasis, local recurrence, or death from any cause) or the last known progression-free date. It was left-truncated for the time of entry into the study if entry was after the start of treatment. The maximum observation time was 3 years. Overall survival (OS_–1TL_) was defined similarly.

Survival rates with 95% confidence intervals (CIs) relative to SHERBOC eligibility status were estimated using the Kaplan–Meier product limit method. If possible, the median survival time with 95% CI was computed using the Brookmeyer and Crowley method [37]. A simple Cox regression analysis with SHERBOC status as the only predictor was performed to obtain an unadjusted hazard ratio (HR) with 95% CI. An adjusted HR for SHERBOC status was estimated using a multiple Cox regression model with the predictors SHERBOC status, age at study entry, tumor grade, ECOG, metastasis pattern, and number of concomitant diseases. The proportional hazards assumptions were checked using the Grambsch and Therneau method [38]. At the time of database closure, there were some patients who went on to the next therapy line without being included in SHERBOC. Some patients moved onto the next therapy line during the conduct of this study. This line would have been the earliest therapy line in which the patients could have been included into the SHERBOC trial. For those patients PFS and OS were also calculated for these patients in relation to SHERBOC eligibility status.

Calculations were carried out using the R system for statistical computing (version 3.0.1; R Development Core Team, Vienna, Austria, 2013).

## Results

### Patient and disease characteristics

**Fig. 1:**
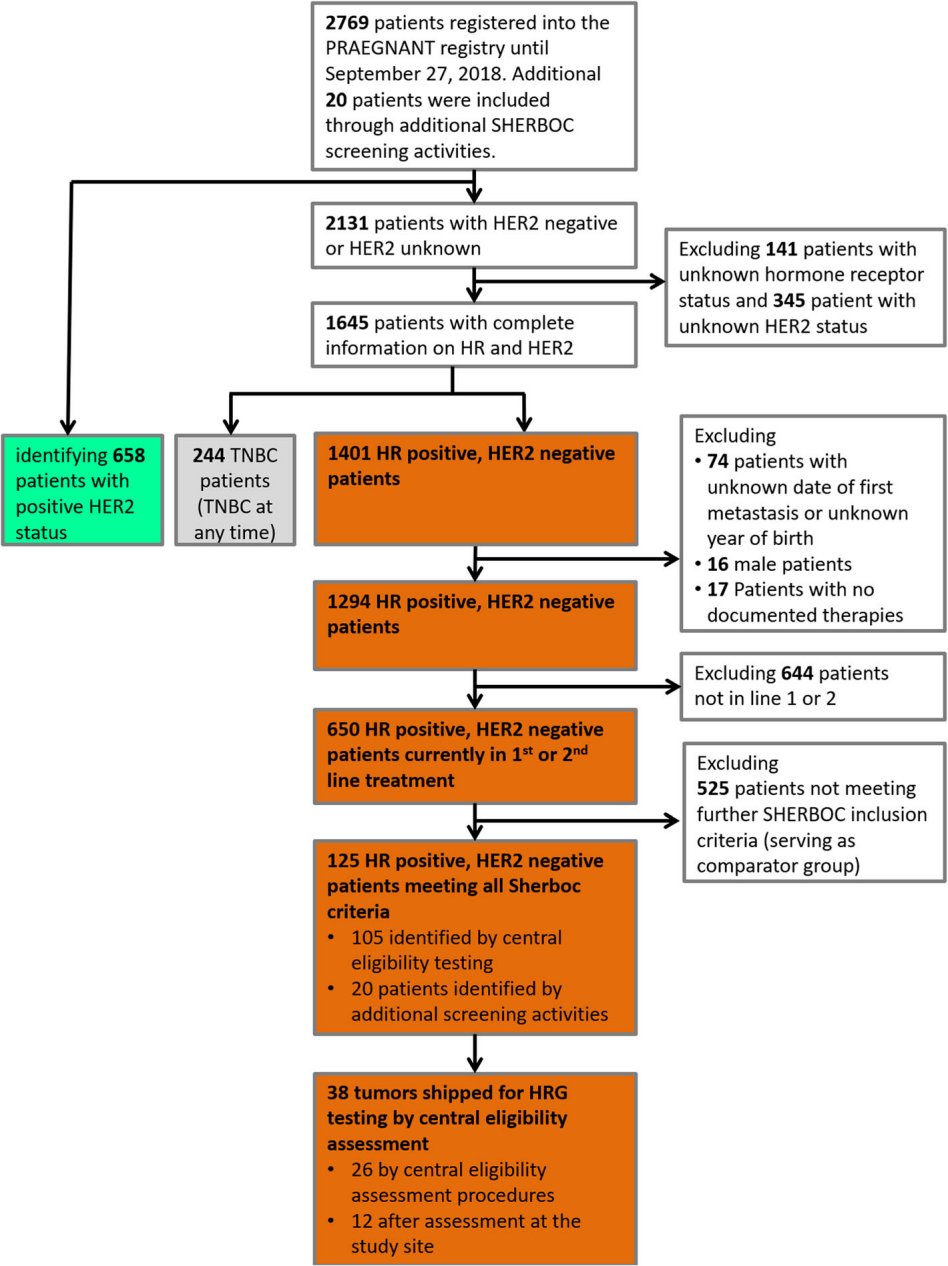
Patient selection

A total of 2769 patients with advanced or metastatic BC were registered in the PRAEGNANT study between July 2014 and September 2018 at 52 study sites. Further 20 patients were included as a result of other SHERBOC screening activities. Patients were excluded in the following hierarchical order: patients with positive HER2 status (*n* = 658), patients with unknown hormone receptor status (*n* = 141), and patients with unknown HER2 status (*n* = 345), leaving 1645 patients with known HER2 and hormone receptor status. Patients with triple-negative tumors (*n* = 244) were excluded. Another 107 patients were excluded because the date of metastasis or their date of birth was not known (*n* = 74), because they were male (*n* = 16), or because therapies were not documented (*n* = 17). The analysis was restricted to patients receiving first-line and second-line treatment. Additional patients were therefore excluded, resulting in 650 patients with HER2-negative, hormone receptor–positive status. Applying the remaining SHERBOC inclusion and exclusion criteria (Additional Table 1) resulted in 125 patients who were considered eligible for the SHERBOC trial and 535 patient who were not eligible. The patient flow chart is shown in Fig. 1. In a subset of 380 of these 650 patients follow-up information was available.

### HRG expression

A total of 38 BC tissue samples were sent to the CAP/ CLIA-certified central laboratory for HRG expression analysis. Of these, 18 (47.4%) were HRG-negative and 14 (36.8%) were HRG-positive (Table 1). The remaining six samples were not evaluable for several reasons (no tumor content, tissue loss on slides, control failure). Patient and tumor characteristics did not differ widely between the HRG-negative and HRG-positive subgroups (Table 2). Interestingly, however, the time from diagnosis to metastasis was 1.8 years shorter in the HRG-positive subgroup compared to patients tested HRG-negative (Table 2). Sample sizes and event numbers were too low for a survival analysis to be conducted relative to HRG status.

**Table 1:** Patient and tumor characteristics in all patients

Characteristic	SHERBOC no(*n* = 525)	SHERBOC yes(*n* = 125)
Age at study entry (years)	60.9 (13.1)	61.1 (11.9)
BMI (kg/m^2^)	26.6 (5.9)	26.0 (5.6)
Time from diagnosis to metastasis (years)	6.0 (6.5)	6.9 (7.5)
Tumor grade		
1	38 (7.8)	13 (12.5)
2	333 (68.4)	75 (72.1)
3	116 (23.8)	16 (15.4)
ECOG score		
0	251 (50.3)	82 (67.8)
1	192 (38.5)	33 (27.3)
≥ 2	56 (11.2)	6 (5.0)
Concomitant diseases		
0 or 1	242 (46.4)	62 (50.4)
2 to 4	192 (36.8)	46 (37.4)
≥ 5	88 (16.9)	15 (12.2)
Metastasis pattern		
Brain	48 (9.2)	4 (3.3)
Visceral	237 (45.3)	42 (34.1)
Bone	88 (16.8)	29 (23.6)
Other	150 (28.7)	48 (39.0)
HRG test		
Negative	–	18 (47.4)^a^
Positive	–	14 (36.8)^a^
Not evaluable	–	6 (15.8)^a^
Ever received chemotherapy		
No	279 (53.1)	125 (100.0)
Yes	246 (46.9)	0 (0.0)
Ever received endocrine therapy		
No	109 (20.8)	12 (9.6)
Yes	416 (79.2)	113 (90.4)
Ever received fulvestrant		
No	356 (67.8)	125 (100.0)
Yes	169 (32.2)	0 (0.0)
Previous CDK4/6i documented		
No	405 (77.1)	4 (3.2)^b^
Yes	120 (22.9)	121 (96.8)
SHERBOC line		
1	290 (55.2)	117 (93.6)
2	235 (44.8)	8 (6.4)

*BMI* Body mass index; *ECOG* Eastern Cooperative Oncology Group; *HRG* Heregulin. *CDK4/6i* CDK4/6 inhibitor

Means and standard deviation (SD) are shown for continuous characteristics, and frequency and percentage for categorical characteristics

^a^ Percentages refer only to the 38 patients for whom a testing was done. For the rest of the population no test results are available due to the early termination of the trial

^b^These patients were confirmed to be clinically eligible for SHERBOC despite CDK4/6i was not documented yet at time of database closure

**Table 2:** Patient and tumor characteristics in patients tested for HRG

Characteristic	HRG-negative (*n* = 18)	HRG-positive (*n* = 14)
Age at study entry (years)	61.6 (15.0)	56.3 (10.1)
BMI (kg/m^2^)	25.6 (4.3)	24.9 (4.2)
Time from diagnosis to metastasis (years)	5.6 (5.8)	3.8 (4.4)
Tumor grade		
1	2 (11.8)	2 (18.2)
2	11 (64.7)	7 (63.6)
3	4 (23.5)	2 (18.2)
ECOG score		
0	13 (76.5)	11 (84.6)
1	4 (23.5)	2 (15.4)
2+	0 (0.0)	0 (0.0)
Concomitant diseases		
0 or 1	8 (44.4)	10 (76.9)
2 to 4	10 (55.6)	2 (15.4)
5+	0 (0.0)	1 (7.7)
Metastasis pattern		
Brain	0 (0.0)	1 (7.1)
Visceral	4 (22.2)	9 (64.3)
Bone	2 (11.1)	1 (7.1)
Other	12 (66.7)	3 (21.4)
Ever received chemotherapy		
No	18 (100.0)	14 (100.0)
Yes	0 (0.0)	0 (0.0)
Ever received endocrine therapy		
No	1 (5.6)	1 (7.1)
Yes	17 (94.4)	13 (92.9)
Ever received fulvestrant		
No	18 (100.0)	14 (100.0)
Yes	0 (0.0)	0 (0.0)
Previous CDK4/6i documented		
No	0 (0.0)	1 (7.1)^a^
Yes	18 (100.0)	13 (92.9)
SHERBOC line		
1	17 (94.4)	12 (85.7)
2	1 (5.6)	2 (14.3)

*BMI* Body mass index; *ECOG* Eastern Cooperative Oncology Group; *HRG* Heregulin. *CDK4/6i* CDK4/6 inhibitor

Means and standard deviation (SD) are shown for continuous characteristics, and frequency and percentage for categorical characteristics

^a^ This patient was confirmed to be clinically eligible for SHERBOC despite CDK4/6i was not documented yet at time of database closure

In the overall international SHERBOC study population (*n* = 194) that was prescreened for HRG, 120 patients (61.9%) were HRG-positive and 60 patients (30.9%) were HRG-negative, while 14 cases (7.2%) were not evaluable.

### Comparison of selected versus nonselected patients

**Fig. 2:**
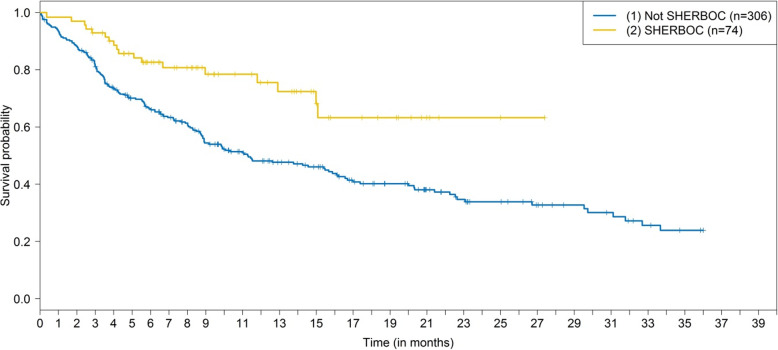
Progression-free survival relative to SHERBOC eligibility status. Kaplan–Meier curves for progression-free survival (PFS_–1TL_) in the therapy line before possible SHERBOC inclusion, relative to SHERBOC eligibility status

**Fig. 3:**
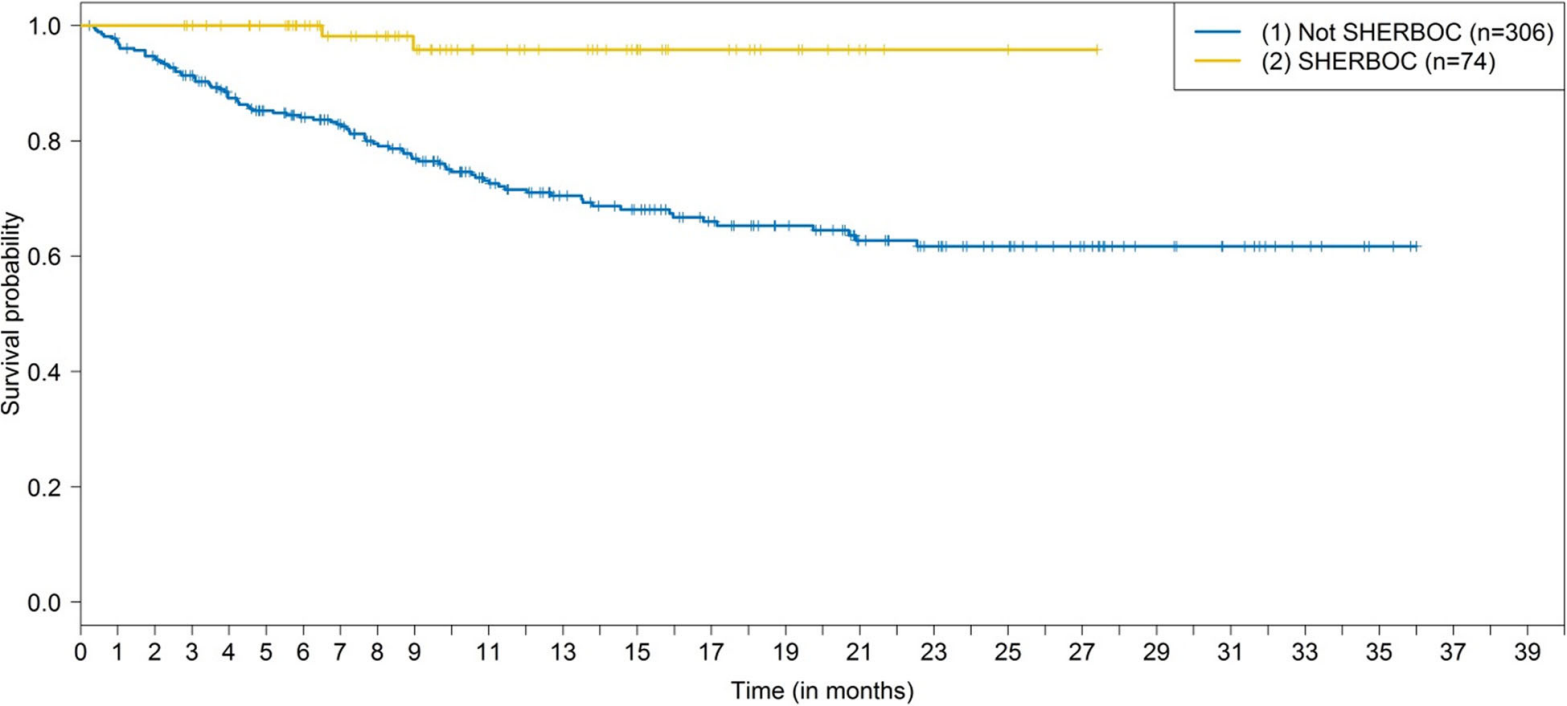
Overall survival relative to SHERBOC eligibility status. Kaplan–Meier curves for overall survival (OS_–1TL_) in the therapy line before possible SHERBOC inclusion, relative to SHERBOC eligibility status

The patient characteristics for selected and non-selected HER2-negative, hormone receptor–positive patients in first-line or second-line treatment are shown in Table 1. With the exception of the number of prior therapies, there were no major differences between the two groups. Age (60.9 vs. 61.1 years), BMI (26.6 vs 26.0) and the number of concomitant diseases were almost identical between the groups (Table 1). Excluded patients appeared to have slightly more aggressive disease, as defined by tumor grade and metastasis, since the time from diagnosis to metastasis was about 1 year shorter in these patients (Table 1). With regard to the prognosis, patients selected for SHERBOC on the basis of clinical parameters (not including HRG testing) had a better PFS_–1TL_ and a better OS_–1TL_ (Tables 3 and 4). The adjusted HR for PFS_–1TL_ was 0.60 (95% CI, 0.36 to 0.98), in favor of patients selected for the SHERBOC trial. The HR for OS was 0.14 (95% CI, 0.03 to 0.59), again in favor of patients selected for the SHERBOC trial. The 2-year PFS rate was 0.34 (0.28, 0.41) for nonselected and 0.63 (0.49, 0.82) for selected patients (Tables 4). The 2-year OS rate was 0.62 (0.55, 0.69) and 0.96 (0.90, 1.00), respectively (Tables 4). The corresponding Kaplan–Meier curves are shown in Figs. 2 and 3. It should be noted, that for this analysis the patients were assigned to the eligible patient group regardless of HRG testing — i.e., HRG-negative patients were included in this analysis. The PFS and OS in the therapy line in which patients would have been included in the SHERBOC trial were similar to the PFS and OS in the previous therapy lines, with patients selected for SHERBOC having a more favorable prognosis (Additional Figs. 1 and 2).

**Table 3:** Unadjusted and adjusted hazard ratios^a^ for SHERBOC status

Outcome	Unadjusted analysis		Adjusted analysis ^b^	
	Hazard ratio (95% CI)	*P* value	Hazard ratio (95% CI)	*P* value
PFS_-1TL_^c^	0.44 (0.27, 0.72)	< 0.01	0.60 (0.36, 0.98)	0.04
OS_-1TL_^c^	0.10 (0.02, 0.40)	< 0.01	0.14 (0.03, 0.59)	< 0.01

*CI* Confidence interval; *HR* Hazard ratio; *OS* Overall survival; *PFS* Progression-free survival

^a^ Reference category is “not SHERBOC”

^b^ HRs are adjusted for age at study entry, tumor grade, Eastern Cooperative Oncology Group grading, metastasis pattern, and number of concomitant diseases

^c^ -1TL refers to the PFS or OS of the therapy line before a possible inclusion into the SHERBOC trial

**Table 4:** Number of events, median survival time, and 6-month, 1-year, and 2-year survival rates, with 95% confidence intervals in the therapy line before a possible inclusion into the SHERBOC trial

	Progression-free survival		Overall survival	
	SHERBOC no	SHERBOC yes	SHERBOC no	SHERBOC yes
At risk	306	74	306	74
Events	169	18	87	2
Median survival time (months)	11.3 (8.9, 16.1)	–	–	–
6-month survival rate	0.66 (0.61, 0.72)	0.83 (0.74, 0.92)	0.84 (0.80, 0.88)	1.00 (1.00, 1.00)
1-year survival rate	0.48 (0.42, 0.55)	0.76 (0.65, 0.88)	0.72 (0.66, 0.77)	0.96 (0.90, 1.00)
2-year survival rate	0.34 (0.28, 0.41)	0.63 (0.49, 0.82)	0.62 (0.55, 0.69)	0.96 (0.90, 1.00)

## Discussion

In this study, a registry for patients with advanced BC was used to identify potential candidates for a specific clinical trial, with a procedure for centralized tumor specimen collection and shipment to a centralized laboratory for HRG evaluation.

With regard to HRG status, these results (36.8% positive cases) are consistent with those of previous studies [39–41]. However, in contrast to the SHERBOC trial, those studies included primary BC cases and patients were not selected and tested for therapeutic approaches. This is the first report of HRG positivity in a cohort of BC patients specifically selected for potential treatment with seribantumab (MM-121). Interestingly, most studies evaluating HRG positivity in BC cases are based on protein expression analyzed using immunohistochemistry. In these studies, the cases varied from having as little as 26% and as much as 84% cytoplasmic HRG-expressing tissue [42, 43]. However, most of them reported expression for approximately 30–50% of the patients analyzed, in line with the HRG positivity rate in the selected SHERBOC cases in this PRAEGNANT study cohort [39–41].

HRG expression has been widely studied in relation to clinical outcomes and it has been proposed as a biomarker for anti-HER3 therapies. In most studies using HRG expression as a biomarker to select responsive breast, ovarian, or lung cancer patients, HRG positivity has solely been defined on the basis of RNA measurements - either with reverse transcriptase polymerase chain reaction (RT-PCR) or RNA-ISH [29, 39, 44]. For example, in a clinical trial evaluating seribantumab (MM-121) in combination with exemestane in patients with ER/PR+, HER2-negative metastatic BC, the expression of prespecified biomarkers (HRG, betacellulin, EGFR, ErbB2, and ErbB3) was analyzed using RT-PCR. In that study, 31% of the patients (17 of 55 evaluated patients) were positive for two or more of these biomarkers, however no detailed positivity rates per biomarker were reported [28]. In patients positive for two of the biomarkers, the HR for PFS was 0.32 (95% CI [0.10–1.00]) [28] Unfortunately, in the PRAEGNANT/SHERBOC cohort the sample size and number of events were too low for a survival analysis to be performed in the 32 patients with available HRG results. Furthermore, it has to be emphasized, that HRG RNA assessment by ISH is a rather new approach for eligibility testing. However, in non-small cell lung cancer patients whose tumors had detectable HRG mRNA expression by ISH, treatment benefit was observed in the seribantumab (MM-121) + erlotinib combination (HR, 0.35; 95% CI, 0.16–0.76;*p* = .008), which indicates suitability of this method for HRG biomarker assessment [45].

Selection of suitable inclusion and exclusion criteria is a crucial process and is decisive for trial success and recruitment rates. In order to elucidate differences between selected and nonselected patients for potential enrollment in SHERBOC, we analyzed patient characteristics and prognoses using data from the PRAEGNANT registry. Among the patients who were not eligible for SHERBOC, almost half were treated with chemotherapy, and this may help explain the differences in outcome between patients who were eligible and ineligible for SHERBOC. It has been reported that the variable “chemotherapy use” is an independent predictor of the prognosis in patients with advanced HER2-negative, hormone receptor–positive BC [46]. This effect was also previously reported in the PRAEGNANT study [47]. It should be noted that in the present study, only patients who were considered eligible or not eligible for SHERBOC were compared, based on clinical parameters and not on HRG test results. The sample size was too small to describe the prognosis in HRG-positive vs. HRG-negative patients. Furthermore, the statements concerning prognosis according to the inclusion/exclusion criteria should be interpreted with caution as the sample size to confirm those findings is too small in patients for whom HRG test results were available.

Another group that should be noted, and which was not eligible for the SHERBOC trial, consists of patients with previous fulvestrant treatment. Approximately one-third of the patients in the PRAEGNANT registry had previously been treated with fulvestrant, which excluded them from enrollment in the SHERBOC trial. However, it has recently been reported that CDK4/6 inhibitors in combination with fulvestrant in early therapy lines result in a clearly improved PFS [48]. An increase in the use of that combination in early therapy lines must therefore be expected, which may have become an increasing recruitment obstacle for this trial. These two examples show that the monitoring of recruitment activities and reasons for exclusion may be very beneficial during the conduct of a clinical trial.

We have reported previously on the use of our registry to identify patients who may be eligible for clinical trials [33], with a sensitivity and specificity of approximately 90% with the comprehensive data capture in the network. Although sensitivity and specificity were not assessed in the current study, there were no inclusion criteria and exclusion criteria that could be considered problematic for applying the query algorithms. Several support systems for clinical trial recruitment have been published and evaluated [49–51]. Most of these are based on electronic health record data and do not use additionally captured data. In the SHERBOC trial, tumor tissue had to be tested for HRG expression. The registry provided an efficient way of using existing data, with access to tumor tissue with the patients’ consent, in order to identify patients who are eligible not only by using clinical inclusion/ exclusion criteria but also by using biomaterials.

Overall, using registries for clinical trial eligibility testing could be a suitable tool in order to improve recruitment numbers and reduce study durations [3]. It further could relieve clinicians and study centers through a centrally managed screening of eligibility and testing of biomarkers. In addition, all patients enrolled in the registry would then be screened for eligibility regardless of whether the patients’ clinic is a study side of the respective trial. Thus, trials would be available for a broader pool of patients and eligible patients could be transferred to a trial site which is not the primary treating clinic. Further, the combination of registries and clinical trials could facilitate research as additional data about clinical history and molecular biomarkers is available within the registry, which might help to better understand prognosis of selected cohorts.

However, also some limitations of registries have to be taken into account. First, conducting a registry study over a long period of time is a quite costly procedure [52]. Continuous study management and monitoring is necessary in order to maintain high standards. Second, for maintaining a registry certain specialists, e.g. data managers or computer scientists, are needed which are usually not part of clinical routine procedures. While the recruitment of trial subjects normally solely relies on physicians and study nurses, for the recruitment using a registry, specialized data mangers are needed in order to provide high data quality and constantly monitor data input. Only a registry with a high quality data can be useful for research or recruitment purpose [53]. Especially completeness of case ascertainment, validity of values for each data point and timelessness of data has been described as quality indicators [53]. Nevertheless, investing into a high quality registry could be of benefit for patients, clinicians, sponsors and stake holders over the long term.

## Conclusion

Overall, this study suggests that a real-world registry for advanced BC could be used for eligibility testing procedures for sophisticated clinical trials, including molecular testing of tumor tissue. This encouraging preliminary experience suggests that patient registries such as PRAEGNANT could be successfully used to enhance patient identification and enrollment in future biomarker-directed cancer treatment trials. Actual efficiency of patient enrollment into a trial assisted by patient selection through a real-world registry and potential benefits resulting from using a registry need to be evaluated in future studies.

## Supplementary Information

**Additional file 1: Table S1.** SHERBOC Study Eligibility Criteria. **Table S2.** Data categories captured in the PRAEGNANT study.

**Additional file 2: Figure S1.** Kaplan–Meier curves for progression-free survival (PFS) starting at the time of possible SHERBOC inclusion, relative to SHERBOC eligibility status. **Figure S2.** Kaplan–Meier curves for overall survival (OS) starting at the time of possible SHERBOC inclusion, relative to SHERBOC eligibility status.

## Data Availability

The datasets used and/or analysed during the current study are available from the corresponding author on reasonable request.

## Abbreviations

–1TL: –1 Therapy line
BC: Breast cancer
CI: Confidence interval
CISH: Competitive in situ hybridization
ER: Estrogen receptor
ET: Endocrine therapy
FFPE: Formalin-fixed, paraffin-embedded
FISH: Fluorescent in situ hybridization
HER: Human epidermal growth factor receptor
HR: Hazard ratio
HRG: Heregulin
IHC: Immunohistochemistry
OS–1TL: Overall survival before inclusion
PFS–1TL: Progression-free survival before inclusion
PR: Progesterone receptor
RNA-ISH: RNA in situ hybridization
RT-PCR: Reverse transcriptase polymerase chain reaction
SD: Standard deviation
SQL: Structured query language
