# Supplementary material for: Heregulin (HRG) assessment for clinical trial eligibility testing in a molecular registry (PRAEGNANT) in Germany
Source: BMC Cancer. 2020 Nov 11;20:1091. doi: 10.1186/s12885-020-07546-1 (PMC7656772; doi:10.1186/s12885-020-07546-1)
Supplement: Supplementary file 1 — Additional file 1: Table S1. SHERBOC Study Eligibility Criteria. Table S2. Data categories captured in the PRAEGNANT study. [file 12885_2020_7546_MOESM1_ESM.docx]

**Additional Table 1 – SHERBOC Study Eligibility Criteria**Inclusion and exclusion criteria in accordance with [[38](#_ENREF_38)].

| Inclusion criteria: To be eligible for participation in the study, patients must meet the following criteria: |
| --- |
| Patients who are HRG-negative do not need to complete screening procedures beyond HRG assessment.  • Patients must have histologically or cytologically confirmed ER+ and/or PR+ (with staining of > 1% cells) breast cancer.  • Patients with confirmed postmenopausal status due to either surgical/natural menopause or ovarian suppression.  • Patients must be HER2-negative.  • Patient must have at least one lesion amenable to either core needle biopsy or fine-needle aspiration.  • Patient must have a positive in situ hybridization (ISH) test for heregulin, as determined by centralized testing of unstained tumor tissue.  • Patients who have progressed following at least one, but no more than two, prior systemic therapies in the locally advanced or metastatic disease setting. One of these therapies must have been based on a CDK4/6 inhibitor.  • Patients with documented progression of locally advanced or metastatic disease as defined by RECISTv1.1 (exception: patients with bone-only metastatic disease are eligible if they have at least two lytic lesions visible on a CT or MRI and have documented disease progression on prior therapy based on the appearance of new lesions).  • Patients with bone-only lesions who have received radiation to those lesions must have documented progression following radiation therapy.  • ECOG performance score (PS) of 0 or 1.  • Patients with adequate bone-marrow reserves.  • Adequate hepatic function.  • Adequate renal function.  • Patient has recovered from clinically significant effects of any prior surgery, radiosurgery, or other antineoplastic therapy.  • Patients who have experienced a venous thromboembolic event within 60 days of signing the main consent form should have been treated with anticoagulants for at least 7 days prior to beginning treatment and for the duration of treatment on this study. |
| Exclusion criteria: patients must meet all the inclusion criteria listed above and have none of the following exclusion criteria: |
| • Prior treatment with an anti-ErbB3 antibody.  • Prior treatment with chemotherapy in the locally advanced or metastatic disease setting.  • Patients cannot have received prior treatment with fulvestrant or other SERDs in the locally advanced or metastatic setting.  • Uncontrolled CNS disease or presence of leptomeningeal disease.  • Inflammatory breast cancer.  • History of another active malignancy that required systemic therapy in the last 2 years. Patients with prior history of in-situ cancer, basal, or squamous cell skin cancer are eligible.  • Patients with an active infection, or unexplained fever > 38.5 °C during screening visits or on the first scheduled day of dosing, which in the investigator’s opinion might compromise the patient’s participation in the trial or affect the study outcome. At the discretion of the investigator, patients with tumor fever may be enrolled.  • Known hypersensitivity to any of the components of seribantumab, fulvestrant, or who have had hypersensitivity reactions to fully human monoclonal antibodies.  • NYHA class III or IV congestive heart failure.  • Patients with a significant history of cardiac disease (i.e., uncontrolled blood pressure, unstable angina, myocardial infarction within 1 year or ventricular arrhythmias requiring medication) are also excluded.  • Uncontrolled infection requiring IV antibiotics, antivirals, or antifungals; or active human immunodeficiency virus (HIV) infection, active hepatitis B infection or active hepatitis C infection. |

CNS, central nervous system; CT, computed tomography; ECOG, Eastern Cooperative Oncology Group; ER, estrogen receptor; HRG, heregulin; MRI, magnetic resonance imaging; NYHA, New York Heart Association; PR, progesterone receptor; SERD, selective estrogen receptor down-regulator.

**Additional Table 2**Data categories captured in the PRAEGNANT study.

| Data continuously captured if applicable | Data assessed at study entry | Data assessed at follow-up care appointments |
| --- | --- | --- |
| Concomitant diseases | Life status, ECOG | Life status, ECOG |
| Concomitant medication | Quality of life | Quality of life |
| Cancer systemic therapies | Breast cancer risk factor questionnaire | Breast and axilla evaluation |
| Cancer radiotherapy | Breast and axilla evaluation | Distant metastasis evaluation |
| Cancer surgery | Distant metastasis evaluation | Biomaterial ascertainment |
| Breast cancer, right side | Biomaterial ascertainment | PRO questionnaires |
| Breast cancer, left side | PRO questionnaires |  |

ECOG, Eastern Cooperative Oncology Group performance status; PRO, patient-reported outcome.
