# Supplementary material for: Heregulin (HRG) assessment for clinical trial eligibility testing in a molecular registry (PRAEGNANT) in Germany
Source: BMC Cancer. 2020 Nov 11;20:1091. doi: 10.1186/s12885-020-07546-1 (PMC7656772; doi:10.1186/s12885-020-07546-1)

**Additional Fig. 1.** Kaplan–Meier curves for progression-free survival (PFS) starting at the time of possible SHERBOC inclusion, relative to SHERBOC eligibility status.


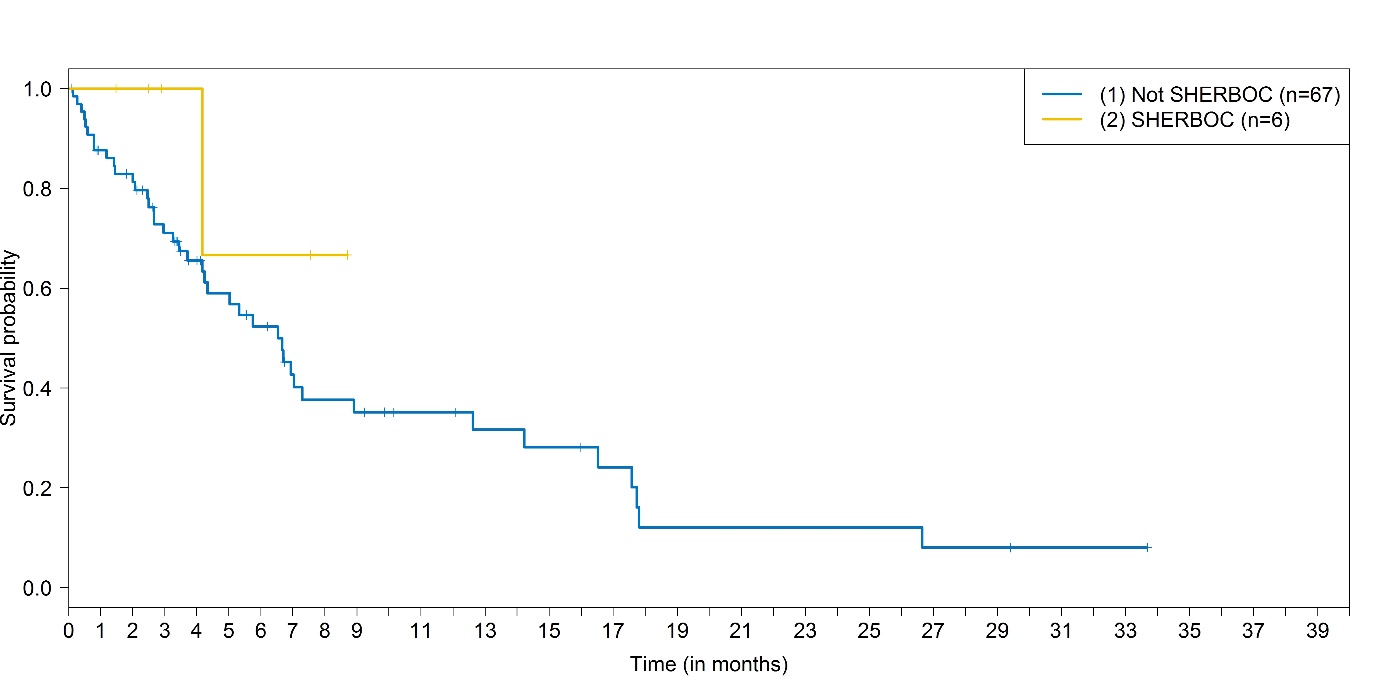


**Additional Fig. 2.** Kaplan–Meier curves for overall survival (OS) starting at the time of possible SHERBOC inclusion, relative to SHERBOC eligibility status.


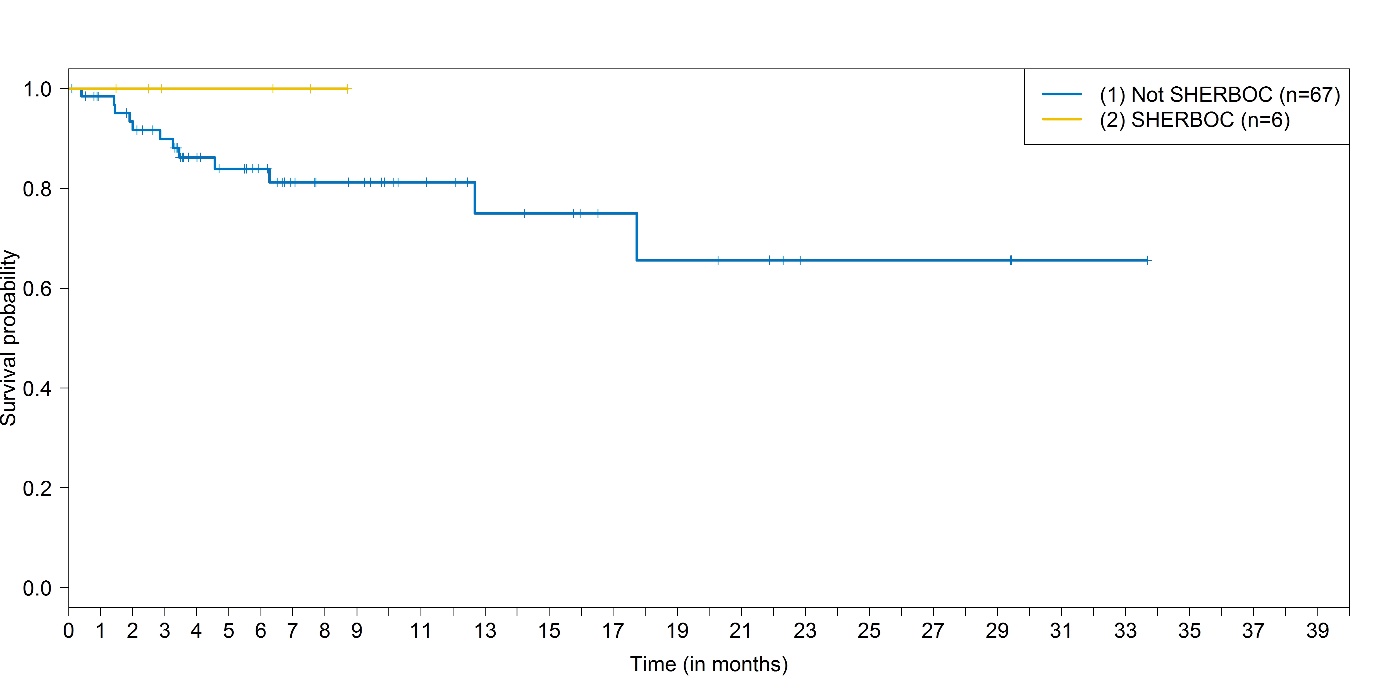

Supplement: Supplementary file 2 — Additional file 2: Figure S1. Kaplan–Meier curves for progression-free survival (PFS) starting at the time of possible SHERBOC inclusion, relative to SHERBOC eligibility status. Figure S2. Kaplan–Meier curves for overall survival (OS) starting at the time of possible SHERBOC inclusion, relative to SHERBOC eligibility status. [file 12885_2020_7546_MOESM2_ESM.docx]
